# Editorial: Autoimmune and inflammatory rheumatic diseases: Identifying biomarkers of response to therapy with biologics: Volume II

**DOI:** 10.3389/fphar.2023.1164723

**Published:** 2023-02-28

**Authors:** Anna Lisa Giuliani, Alessandra Bortoluzzi, Francesca Oliviero

**Affiliations:** ^1^ Department of Medical Sciences, University of Ferrara, Ferrara, Italy; ^2^ Department of Medicine, DIMED, University of Padua, Padua, Veneto, Italy

**Keywords:** autoimmune diseases, rheumatic diseases, inflammation, biologic therapies, biomarkers

Abnormal immune responses against tissues and organs of the body including cell death dysregulation, uncontrolled complement cascade activation, inflammation, recruitment of self-reactive immune cells leading to uncontrolled release of cytokines and autoantibodies represent the basis of a wide variety of systemic autoimmune diseases (SADs), rheumatic diseases included. In most cases, the mechanisms underlying these diseases are not clearly identified and a great number of studies is aimed at their elucidation (Furini et al., 2019; Liu et al., 2021; Lundberg et al., 2021; Zucchi et al., 2022).

The identification of markers, such as specific cytokines or growth factors, or characteristic antibodies, represent an urgent need for disease classification and severity prediction (Yu et al., 2021). In line with that, a study from Zhou J et al. on patients affected by Idiopathic Inflammatory Myopathies (IIMs), found increased levels of various cytokines (Eotaxin, IL-7, IL-18, IP10, MCP1, MCSF, MIG, and SCGFβ), and correlation of levels of some of these cytokines with clinical indices. Additionally, groups of patients expressing different myositis specific antibodies (MSA), namely, anti-ARS, anti-MDA-5, and anti-TIF1γ, which are used for diagnosis and classification of IIMs, presented unique cytokine expression patterns (Front Pharmacol. 2022 April 20; 13:852055).

During the last few years, the pharmacological treatments of these diseases have included an increasing number of various biological drugs ranging from monoclonal antibodies and small inhibitory molecules directed against cytokines or their cell receptors, to therapies targeted to key elements and regulators of cellular responses (e.g., jak-inhibitors) (Khoo and Limaye, 2020; Tanaka, 2020; Huang et al., 2021; Thoreau et al., 2021; Singh, 2022).

In contrast to traditional immunosuppressive strategies, these drugs show selectivity of action towards specific targets and are currently employed to induce remission or treat specific organ involvements. One of the main treatments based on biological drugs is represented by the subcutaneous administration of tumour necrosis factor inhibitors (SC-TNFis) that has turned out to be of great relevance in the treatment of chronic progressive immune mediated rheumatic diseases (IMRDs) such as rheumatoid arthritis (RA), psoriatic arthritis (PsA), and ankylosing spondylitis (AS). Altogether IMRDs are responsible of joint deformation and progressive disability that can impact in healthcare resource utilization (HCRU) costs. In their work, Carballo N et al. have evaluated the impact on HCRU costs of persistent and non-persistent SC-TNFis therapy comparing costs between 12 months before and 12 months after the beginning of treatment. The results show that long-term persistent treatment of IMRD naïve patients with SC-TNFis may indeed be associated with savings in HCRU costs (Front Pharmacol. 2021 November 29; 12:752879), indicating that targeted, and possibly personalized therapies, may have economic significance, besides being undoubtedly relevant to patients’ wellbeing.

Among SADs, systemic lupus erythematosus (SLE), Sjogren’s syndrome (SS), and antiphospholipid syndrome (APS) are important mimickers of multiple sclerosis (MS) since invariably affecting the CNS with various MS-like manifestations. A consistent differentiation between MS and CNS involvement in the setting of SADs is not fully achievable neither by brain MRI, cerebrospinal fluid (CSF) analysis, nor by autoantibodies (i.e., ANA or antiphospholipid antibodies) detection. The study from Karathanasis D. K. et al. showed that some distinct clinical, imaging, and laboratory characteristics may be helpful in early differentiation between MS and CNS-involving SADs, and that peripheral blood type I IFN activity was a strong predictor for SADs presenting with CNS involvement. These results allow for optimal therapeutic strategies, particularly considering the potentially significant therapeutic role of type I IFN receptor blockade in SAD patients with evidence of CNS involvement as a cardinal manifestation (Front Pharmacol. 2022 August 12; 13:898049).

In some cases, these innovative drugs have unfortunately failed to meet their primary endpoints in randomized-controlled trials, especially in complex diseases such as SLE and systemic sclerosis (SSc). This has led to changes to therapies currently used as well as to the development of new approaches in some cases based on the use of natural ingredients whose mechanisms of action have become more known (Wang et al., 2021).

Osteoarthritis (OA) is one of the most common joint degenerative diseases in the world (Abramoff and Caldera, 2020; Oliviero et al., 2022). In the treatment of OA novel reagents such as IL-1 antagonists and nerve growth factor inhibitors have entered clinical trials. Additionally, increasing evidence demonstrated that active ingredients of natural plants have great potential for treating this disease. Among these ingredients, in their review (Front Pharmacol. 2022; November 17; 13:945876) Shentu et al. explored the pharmacological effects of bioactive alkaloids obtained from traditional Chinese medicines, such as Matrine and Sinomenine, and some other products of plant extracts such as Osthole, and Curcumin, Loganin and Morroniside, which are significant iridoid glycosides, the yellow flavonoid Quercitrin, Resveratrol, Ligustilide, Icariin, Baicalein, and modin. Moreover, the authors suggest that use of novel drug delivery strategies may overcome the shortcomings of conventional preparations and enhance the bioavailability of drugs, as well as decrease significantly the side effects (Front Pharmacol 2022; November 17; 13:945876). As regard resveratrol, it has long been regarded as a potential antioxidant drug for RA treatment. The review from Sheng et al. (Front Pharmacol. 2022 August 22; 13:829677) treats of how resveratrol is currently considered to exert therapeutic effects on RA by activating silent information regulator 1 (SIRT1) and its downstream pathways. There is notable crosstalk between the SIRT1 and NF-κB pathways, and these pathways, which play an essential role in the development of RA, are unexpectedly linked to the influence of resveratrol.

In conclusion, in this Research Topic some new relevant aspects regarding pathogenetic mechanisms, new treatment strategies and identification of biomarkers in systemic autoimmune or degenerative diseases have been explored.

## References

[B1] Abramoff B. Caldera F. E. (2020). Osteoarthritis: Pathology, diagnosis, and treatment options. Med. Clin. North Am. 104(2), 293–311. 10.1016/j.mcna.2019.10.007 32035570

[B2] Furini F. Giuliani A. L. Parlati M. E. Govoni M. Di Virgilio F. Bortoluzzi A. (2019). P2X7 receptor expression in patients with serositis related to systemic lupus erythematosus. Front. Pharmacol. 10, 435. 10.3389/fphar.2019.00435 31110478 PMC6501575

[B3] Huang J. Fu X. Chen X. Li Z. Huang Y. Liang C. (2021). Promising therapeutic targets for treatment of rheumatoid arthritis. Front. Immunol. 12, 686155. 10.3389/fimmu.2021.686155 34305919 PMC8299711

[B4] Khoo T. Limaye V. (2020). Biologic therapy in the idiopathic inflammatory myopathies. Rheumatol. Int. 40 (2), 191–205. 10.1007/s00296-019-04467-6 31680207

[B5] Liu L. Yuan Y. Zhang S. Xu J. Zou J. (2021). Osteoimmunological insights into the pathogenesis of ankylosing spondylitis. J. Cell Physiol. 236 (9), 6090–6100. 10.1002/jcp.30313 33559242

[B6] Lundberg I. E. Fujimoto M. Vencovsky J. Aggarwal R. Holmqvist M. Christopher-Stine L. (2021). Idiopathic inflammatory myopathies. Nat. Rev. Dis. Prim. 7 (1), 86. 10.1038/s41572-021-00321-x 34857798

[B7] Oliviero F. Scanu A. Galozzi P. Ramonda R. (2022). Synovial fluid analysis to identify osteoarthritis. J. Vis. Exp. (188). 10.3791/64351 36342180

[B8] Singh J. A. (2022). Treatment guidelines in rheumatoid arthritis. Rheum. Dis. Clin. North Am. 48 (3), 679–689. 10.1016/j.rdc.2022.03.005 35953230

[B9] Tanaka Y. (2020). State-of-the-art treatment of systemic lupus erythematosus. Int. J. Rheum. Dis. 23 (4), 465–471. 10.1111/1756-185X.13817 32134201 PMC7187183

[B10] Thoreau B. Chaigne B. Renaud A. Mouthon L. (2021). Treatment of systemic sclerosis. Presse Med. 50 (1), 104088. 10.1016/j.lpm.2021.104088 34718115

[B11] Wang Y. Chen S. Du K. Liang C. Wang S. Owusu Boadi E. (2021). Traditional herbal medicine: Therapeutic potential in rheumatoid arthritis. J. Ethnopharmacol. 279, 114368. 10.1016/j.jep.2021.114368 34197960

[B12] Yu H. Nagafuchi Y. Fujio K. (2021). Association between XRCC3 Thr241Met polymorphism and risk of gynecological malignancies: A meta-analysis. Biomolecules 254-255 (7), 11–17. 10.1016/j.cancergen.2021.01.003 33515810

[B13] Zucchi D. Elefante E. Schiliro D. Signorini V. Trentin F. Bortoluzzi A. (2022). One year in review 2022: Systemic lupus erythematosus. Clin. Exp. Rheumatol. 40 (1), 4–14. 10.55563/clinexprheumatol/nolysy 35088691

